# “Overestimated technology – underestimated consequences” – reflections on risks, ethical conflicts, and social disparities in the handling of non-invasive prenatal tests (NIPTs)

**DOI:** 10.1007/s11019-023-10143-1

**Published:** 2023-03-18

**Authors:** Marion Baldus

**Affiliations:** Faculty of Social Work, Hochschule Mannheim / Mannheim University of Applied Sciences, Paul-Wittsack-Str. 10, Mannheim, Germany

**Keywords:** Implementation of NIPT, Marketing and econimization, Ethical conflicts, New responsibilities, Critical thinking

## Abstract

New technologies create new complexities. Since non-invasive prenatal tests (NIPTs) were first introduced, keeping pace with complexity constitutes an ongoing task for medical societies, politics, and practice. NIPTs analyse the chromosomes of the fetus from a small blood sample. Initially, NIPTs were targeted at detecting trisomy 21 (Down syndrome): meanwhile there are sequencing techniques capable of analysing the entire genome of the unborn child. These yield findings of unclear relevance for the child’s future life, resulting in new responsibility structures and dilemmas for the parents-to-be.

The industry’s marketing strategies overemphasize the benefits of the tests while disregarding their consequences. This paper chooses the opposite path: starting with the underestimated consequences, it focuses on adverse developments and downsides. Disparities, paradoxes, and risks associated with NIPTs are illustrated, ethical conflicts described. Indications that new technologies developed to solve problems create new ones are examined. In the sense of critical thinking, seemingly robust knowledge is scrutinized for uncertainties and ambiguities. It analyses how the interplay between genetic knowledge and social discourse results in new dimensions of responsibility not only for parents-to-be, but also for decision-makers, authorities, and professional societies, illustrated by a review of different national policies and implementation programmes. As shown by the new NIPT policy in Norway, the consequences can be startling. Finally, a lawsuit in the United States illustrates how an agency can risk forfeiting its legitimation in connection with the inaccuracy of NIPTs.

## Market launch and implementation of NIPT in Europe and the USA

Prenatal diagnostic methods have been described in health care as a way to promise a sense of security and control in the period leading up to parenthood. Methods that examine the unborn child without putting it at risk are highly accepted, which is a prime reason why the introduction of non-invasive prenatal tests (NIPTs) in 2011 aroused global interest. The tests can be carried out at an early stage of pregnancy, they are risk-free and low-threshold, as only a small blood sample is needed. It has meanwhile become a standard part of the medical repertoire in over sixty countries around the world (Ravitsky et al. 2021; Holloway et al. 2022) – despite major controversies since its introduction and despite a growing number of reliability concerns (FDA 2022, Wise 2022).

NIPT technology was first released in Hong Kong in 2011 and shortly after was introduced commercially in the United States (US) (Allyse et al. 2015, 114). 30% of all NIPT companies are based in the US, “a significantly larger percentage than is found in any other countries” (Holloway et al. 2022: 52). Sales of the tests have grown continuously since the first launch onto the market, reaching a total of 788 million USD in 2021, and predicted by the Market Study Report to rise by a further 13.9% annually between 2022 and 2030 (GlobeNewswire 2022).

NIPTs were initially rolled out as a healthcare product to be paid for by patients privately, with the pregnant woman ordering the test individually through a medical provider. Marketing strategies addressed potential customers directly, promising them relief from concern, security, and freedom from risks for the unborn child. Following this path, the tests rapidly spread throughout the USA, China und Europe as a self-payer service, with the active promotion, marketing, and evaluation being taken care of by the manufacturers of the tests themselves. A few years later, the first European countries gradually adopted the implementation of NIPTs and initiated programmes for the financing of the tests by the public healthcare system (Ravitsky et al. 2021).

Switzerland was the first country to offer such a programme, refinancing the costs of the non-invasive risk appraisal for trisomies 21 (Down syndrome), 18 (Edwards syndrome), and 13 (Patau syndrome) via the “obligatory healthcare insurance programme” since July 2015 (Ochsenbein-Kölble et al. 2018). The cut-off here is a defined risk threshold that is calculated from the first-semester test (FST), which is a standard service offered in Switzerland. If the FST yields a risk score lower than the threshold of 1:1000, the pregnant woman is entitled to an NIPT financed by the health insurance (HI) scheme. The objective of this approach is to avoid invasive testing procedures and use them only when the nonspecific risk assessment of the FST is confirmed by the more specific NIPT result. In this case, NIPTs serve as an intermediate step to more precisely define an initial result – which depending on the circumstances may be a false-positive – by means of a second procedure that does not jeopardize the pregnancy. Invasive procedures are associated with a risk of miscarriage of between 0.5% and 2% according to Kolleck and Sauter (2019). Steinfort et al. (2021), who conducted a multicenter, retrospective cohort study over an 11-year period, found a procedure-related risk of miscarriage after mid-trimester amniocentesis of less than 0.4%. Other reviews suggest the number is even lower, depending on the training and experience of medical staff (Salomon et al. 2019).

If the NIPT yields a high probability for a chromosomal defect, the next step is an invasive follow-up examination to confirm or refute the result. We must always remember: NIPT technology is not a diagnostic procedure – it merely calculates probabilities. Positive results require confirmation via invasive diagnostic testing. This is a fact that the test manufacturers’ information leaflets have failed to communicate clearly in the past (Skirton et al. 2015, Nuffield Council on Bioethics 2017, Holloway et al. 2022); instead, they promote the tests as being “reliable, accurate and offering peace of mind” (Han 2022; Holloway et al. 2022: 54). The Nuffield Council on Bioethics guidelines (2017) as well as a recent HASTING CENTER REPORT (Holloway et al. 2022) address this imbalance which is of concern because women might be misled through biased communications from NIPT companies: “Poor-quality information poses the potential to harm from increased shock, distress, and confusion upon receipt of a high-chance result and may even lead to termination of an unaffected fetus if the possibility of a false-positive result is not clearly communicated” (Holloway et al. 2022: 51).

De facto, the clinical validity of NIPTs varies considerably depending on the age of the pregnant woman and on the chromosomal anomaly in question. So far, NIPT technology can be appraised as a “robust” method solely for trisomy 21 with a positive predictive value (PPV) of 80% (Hu et al. 2019: 5; Kolleck and Sauter 2019). The PPV for trisomies 18 and 13, which are also included in all programmes financed by HI schemes, is substantially poorer; it is 60% for trisomy 18 and slightly below 15% for trisomy 13 (Hu et al. 2019: 5). In the case of other syndromes, e.g. Turner syndrome (monosomy X), at just approx. 9% it is much lower still (Kolleck and Sauter 2019). Thus, getting a result with a high probability of a particular condition does not mean that the fetus is affected. Examining the PPV for an individual case is highly complex; parameters such as pre-existing risks, individual age, and the age-specific prevalence of various chromosomal conditions must be considered. Another reason for the limitations of the accuracy of NIPTs is the fact that the test cannot clearly distinguish between fetal DNA fragments on the one hand and other DNA fragments that occur only in the placenta on the other. Placental mosaicism, the demise of a twin, or an undetected maternal malignancy may also be the cause of discordant results (Benn et al. 2019). This is due to the fact that the “tumor DNA contains multiple areas of duplications and deletions across the genome” (ibid.: 339). In this case, not the fetus but the mother would be affected by a health problem. When interpreting and discussing a positive NIPT result, all these different aspects must be taken into account. This makes it even more complex to interpret positive NIPT results.

Thus, the indication of a trisomy or other genetic mutation in the unborn child may represent a false-positive result. Therefore, it is important to always clarify a positive result by invasive diagnostics. When many tests are performed, the number of positive results that need to be followed up increases, and the set goal of minimizing the number of invasive procedures overall may not be achieved. Data from Switzerland clearly show this correlation: the number of amniocenteses has dropped only slightly following the implementation of NIPTs – from 57 to 48% in the group of women with high-risk pregnancies (Vinante et al. 2018). As a consequence, the effect wished for by many women, i.e. the avoidance of having to undergo an invasive examination and thus jeopardizing the pregnancy, is thwarted to a substantial degree. If NIPTs are widely used, a paradox effect occurs: the number of invasive examinations rises. This phenomenon is even more significant in screening programmes that offer genome-wide testing to all pregnant women regardless of their a priori risk, as is the practice in the TRIDENT-2 study in the Netherlands. Genome-wide tests performed as part of this screening provide additional results, some of which are of unknown origin. Further examinations follow, so the total number of invasive diagnoses increases: “Screening for additional findings has inevitably led to an increased number of invasive tests performed” (van Prooyen Schuurman et al. 2022, 1148). The paradox effect only occurred in TRIDENT-2, whereas in the first study, TRIDENT-1, “the number of invasive procedures showed a remarkable reduction” (Bilardo 2021: 941). This is because the NIPT in TRIDENT-1 was exclusively offered to women at increased risk and restricted to a small scope of chromosomal variations (ibid.).

Countries such as Belgium, the Netherlands, Sweden, Lithuania, Italy, Greece, United Kingdom etc. soon followed in the footsteps of the “trailblazer” Switzerland. Currently there are 17 European countries in which NIPT technology has been adopted as a part of their publicly financed prenatal care systems. Regulations regarding the scope of testing, test products, consultation processes, financing, personal financial contributions, and inclusion criteria vary substantially country to country and follow entirely different logical precepts (Gadsbøll et al. 2020; Ravitsky et al. 2021; Reinsperger 2022).

Following an exhaustive process of consultation and hearings within the Gemeinsamer Bundesauschuss (G-BA, Federal Joint Committee), Germany too finally passed the resolution for the financing of such tests by the statutory HI funds, ratified since 1 July 2022 (G-BA 2019a). Since this date, pregnant women are entitled to testing for trisomies 21, 18, and 13. The resolution was preceded by a three-year process in which the methods were validated, one that was accompanied by vehement criticism within professional circles and in particular associations for the welfare of people with disabilities (Kolleck and Sauter 2019). In contrast to Switzerland, the German model does not specify a cut-off above which financing is provided by the HI funds. This is due on the one hand to the fact that the FST is not a standard procedure in Germany, and on the other because the intention is to avoid giving the impression that NIPT is a routine examination that automatically applies for all high-risk pregnancies. Rather the physician him-/herself can decide in each specific case whether the pregnancy is one “that urgently requires further supervision” (G-BA 2019a). What exactly is meant by a pregnancy urgently requiring further supervision has not, however, been conclusively defined. The information leaflet for insurees that was written on behalf of the G-BA and is designed as an information handout for pregnant women ultimately opted for the following formulation: “The costs will be reimbursed when other examinations have yielded an indication of trisomy [or *inserted by author*] when a woman together with her physician comes to the conviction that the test is necessary in the light of her personal circumstances. This situation may arise when the possibility of trisomy is such a burden for the woman that she wishes to clarify the risk conclusively” (G-BA 2021). Just how this aspect of the mutual “conviction” of the “necessity” of the test can be achieved in the practical setting remains undefined. Doubek, President of the Berufsverband der Frauenärzte (BVF, Professional Association of Gynaecologists) in Germany, thus speaks of an “entirely new situation” in the area of consultation for NIPTs and a “far-reaching paradigm shift” (Richter-Kuhlmann 2022). This shift lies in the fact that now in principle each and every pregnant woman has access to an NIPT as a service covered by medical insurance: “Put simply, this means that if a pregnant woman claims that she has substantial fears or concerns, then these subjective fears or concerns are sufficient grounds for the indication and/or basis for action to carry out the NIPT” (Doubek, quoted after Richter-Kuhlmann 2022).

The situation in Poland, by contrast, is completely different: on the one hand, access to genetic prenatal diagnostic procedures “is defined as one of the most important instruments of self-determination of women in the area of reproduction” (Steger, quoted after Klein 2022), while on the other hand barriers have been erected that restrict access to free-of-charge PND (prenatal diagnostics). This is due to a “conscience clause” that permits the physicians in charge of treatment to themselves decide “whether or not to offer PND” (ibid.). Religion and the church here exert a direct influence on the provision and use of prenatal examinations. “This has meant that in recent years many prenatal examinations have been denied to women, since examinations of this type cannot be reconciled with the physicians’ conscience or faith” (ibid.). The same also applies to the issue of women’s access to abortion; the access was further restricted by the reform of the abortion laws in October 2020. Since this time, with very few exceptions the termination of pregnancy counts as an unconstitutional act. While the diagnosis of a disability or illness in the fetus per se does not constitute an exception (Kortas 2022), a medical certificate confirming that the continued pregnancy may jeopardize the woman’s mental health and her life may be interpreted as such. However, obtaining such a medically indicated certificate is very difficult in practice; most physicians refrain from issuing such a certificate “out of fear of forfeiting their career” (ibid.) and of potential legal consequences.

### Unequal access – dual system vs. uniform system

Most countries that have integrated NIPTs into their national screening programmes have established a dual system: parallel to the HI-funded system for a subgroup of pregnant women, there is a private sector that provides all other pregnant women – independent of their age and other risk parameters – access to the tests on a self-payer basis.

The situation in the United States is similar. Almost all private health insurance funds offer the reimbursement of NIPTs; in the publicly sponsored Medicaid programmes, this differs from state to state and is based on criteria such as age, risk profile, and test spectrum. In nine US states, however, NIPTs are not reimbursed by Medicaid (Gadsbøll et al. 2020). The tests can be carried out at people’s own expense in all states, and this option is used on a large scale. According to estimates, between 28% and 50% of all pregnant women in the USA take recourse to a NIPT (Gadsbøll et al. 2020).

In Europe, usage rates are estimated to be about 15% in Italy, 10–35% in Switzerland, 20% in Norway, 30% in Germany, 51% in the Netherlands (Ravitsky et al. 2021), and 75% in Belgium (Gadsbøll et al. 2020). The fact that the Netherlands and Belgium have the highest rates is due to the so-called “first-line” or “first-tier” approach that these two countries have adopted: testing is performed for all pregnant women who consent regardless of a priori risks; the programme in the Netherlands is scientifically accompanied in the form of the TRIDENT II study (Bilardo et al. 2021, van Prooyen Schuurman et al. 2022). This far-reaching uniform screening approach “is perceived as unconventional for the traditionally cautious Dutch system” (Bilardo 2021: 941). In the past the Netherlands used to be careful and cautious in implementing screenings. A law called the “Population Screening Act” (ibid.) regulates the implementation of new testing. The aim of the law is to protect citizens against negative effects or side-effects of screening. Therefore, tests may not be marketed without a license from the Dutch government (ibid.). In 2014, the state license for TRIDENT-1 was granted (ibid.). Three years later, a second license was granted for TRIDENT-2 (ibid.). Since then, the Netherlands have become one of the countries that perform NIPTs most frequently.

### From the private sector to state programmes

In a nutshell, NIPT technology can be described as a low-threshold and globally usable method that was developed by commercial companies as so-called laboratory-developed tests (LDTs) and initially launched in the private healthcare sector (Ravitsky et al. 2021). Within just a few years, the method was then successively adopted into nationally sponsored screening programmes with very differently defined access criteria and infrastructures (Ravitsky et al. 2021; Reinsperger 2022). In Europe, this process has meanwhile progressed to a very high level. While ethical and social issues that relate to the NIPT technology and its use have already been discussed in most countries, this has done little to contain the spread of the tests over time. Even in countries in which social and ethical implications were initially subjected to critical debate (United Kingdom, Germany, the Netherlands) the use of the tests has become widespread. One exception here is Norway, a country that is known for its relatively restrictive attitude towards prenatal diagnosis (Salvesen et al. 2022; Magelssen et al. 2018). Long after its Scandinavian neighbours started implementing the NIPT technology, Norway still tightly adhered to a strict ʻNo-NIPT policyʼ.

### Norway as an example: from the ideal of a society with “room for all” to a “sorting society”?

“An important principle in Norway is the ideal of a society in which everyone has their own place, independent of whether they were born with a specific need or suffer from serious disorders” (Bioteknologiradet 2020, Levold et al. 2021). At the same time, the ideal of a society with “room for all” conflicts with women’s reproductive rights and their right to information and self-determination; rights that are accorded a very high priority in Norway. This ambivalence, which becomes apparent at the intersect between the reproductive autonomy of women and the right of handicapped people to their recognition and dignity, was clearly addressed in the explanatory notes of the Biotechnology Council regarding the revision of the Biotechnology Act in 2020: “The provision of tests by the public sector, linked with the pregnant woman’s right to self-determination, to this extent can collide with the principle that society should offer room for all” (ibid.). There are voices that fear that selective pregnancy terminations following abnormal findings will ultimately lead to a “sorting society” (Magelssen et al. 2018: 2, Levold et al. 2021: 9) and place this in an associative context on a level reminiscent of “eugenic selection practices in Nazi Germany” (ibid.). The possibility of a modification of the mindset of future parents to welcome each and every newborn child in the direction of a “*sorting* mindset” (ibid.) is also thematized as a potential problematic consequence.

The discussion about fetal diagnosis in the ambivalence between providing treatment/medical help to women and the fetus on the one hand and sorting options offered by these tests on the other hand has a long tradition in Norway. It was re-ignited by the political controversy surrounding NIPT from 2012 onwards, eventually leading to a more liberal regulation (Levold et al. 2021). In Norway, the ‘frame’ sorting society is based on a Christian humanistic ethical rationality and refers primarily to the “sorting option” that NIPT offers (Levold et al. 2021: 13). This “sorting frame” is contrasted with the “autonomy/treatment frame” (ibid.: 9), which emphasizes the possible treatment options and the woman’s right to self-determination (ibid.: 13). As Levold et al. (2021) have shown, balancing these framings has been a very fragile and controversial political process over the years. A salient point in this controversy was the argument that the purpose of introducing NIPT screening in Norway was not to identify fetuses with trisomies in order to screen them out, but to reduce the number of invasive procedures and the associated risks of miscarriage (ibid.: 16).

Salvesen et al. (2022: 579) draw attention to another controversial issue: cost calculations that set off expenses against benefits. In the case of NIPTs, these are the costs of the screening programmes compared to the costs saved by the prevention of the birth of children diagnosed with Down syndrome. “Given that the ʻbenefitʼ of NIPT is termination of pregnancy, it is ethically challenging to calculate saved costs for avoiding the birth of a baby with Down syndrome in a country ranking on top of the prosperity index because of the freedom it offers its citizens, the quality of healthcare system and social bonds between its people. Can we make these calculations in a society ʻwith room for allʼ?“ (Salvesen et al. 2022: 579) the authors ask. These considerations and reflections in the Norwegian debate also play a role in other countries, but are communicated less candidly in terms of ambivalences and inconsistencies to social principles and ideals. However, cost-effectiveness analyses and prevention models have a long history in several countries and can be traced back to 1973, shortly after the first prenatal detection of trisomy 21 was made (Stein, Susser, Guterman 1973: 306).

A look at Norway is interesting from another viewpoint as well: Norway is a country that – in contrast to Poland – stands for a very liberal attitude towards abortion. Nevertheless, in the eyes of the majority of the population an abortion after an anomalous diagnostic result constitutes an entirely different issue, in particular regarding the question whether it is justifiable to terminate a pregnancy after the diagnosis of trisomy 21 (Down syndrome): “In spite of a strong support for the Norwegian abortion law including abortion on demand, the Norwegian public tends to regard abortion after prenatal diagnosis as something different. [ ] The closer you get to the diagnosis of Down syndrome the more controversial it becomes” (Magelssen et al. 2018: 7). Despite this controversy, the majority of would-be parents in Norway chooses abortion following the diagnosis of Down syndrome; similar to most other European countries, the rate in Norway is about 90% (Magelssen et al. 2018: 2).

Up until 2017, Norway adhered to a very reserved attitude towards prenatal diagnostics. Screening included neither an ultrasound scan in the first trimester nor the performance of an NIPT; NIPT technology was not available anyway, neither on a private basis nor as an HI-funded service. The first exceptions to this policy were created in 2017: an opening clause provided women aged 38 and older and women with a raised risk with access to NIPT technology as a “second-tier test” (Salvesen et al. 2022: 577). NIPTs remained unavailable to all other pregnant women, even via the private sector, simply because there was no private sector. If women wished to take an NIPT outside the opening clause, they could accomplish this only by travelling beyond the borders of the country (to Sweden or Denmark). The revision of the Biotechnology Act in 2020 led to a radical change in the situation.

### The biotechnology act 2020 in Norway

The Biotechnology Act 2020 was passed in 2020. It became far more liberal than assumed by the government (Levold et al. 2021: 19) and expected by agencies being part of this fragile and turbulent process (ibid.: 6). Since then, following a legally required consultation with a midwife or physician every pregnant woman is entitled to take a NIPT for trisomies 21, 18, and 13. For women under the age of 35, this is possible only on the basis of a private patient (700€ to 800€, including ultrasound); for women aged 35 and older or with a raised risk, the costs are reimbursed by the statutory HI funds (Bioteknologiradet 2020). Another innovation is that for the first time it is now possible to establish private service centres for ultrasound and NIPTs. The precondition for opening such a centre is the passing of a certification process (ibid.). The first centres opened their doors in the summer of 2021. Since then, Norway has had a dual system leading to unexpected consequences in public health centers.

### Migration of technical personnel to the private sector

The establishment of private centres swiftly led to a migration of medical professionals from state-funded hospitals to the private sector. Salvesen et al. (2022: 578) speak of a “brain drain”. Glad describes the situation as follows: “Highly specialised healthcare personnel have chosen to take up more appealing positions in the private health service rather than work at public maternity clinics” (Sitras – personal communication April 2022, quoted after Glad 2022: n.p.).

The establishment of two parallel systems took place “despite robust counterarguments and warnings from medical communities” (Glad 2022: n.p.). This is now jeopardizing the infrastructure in the public sector; many hospitals in the northern regions of the country in particular are now at existential risk (ibid.). As a measure to stop this trend, Glad calls for action “to eliminate the market basis for private actors and help curb the loss of competence in the health trusts” (ibid.)

### Equal access or a divide of women?

This proposal for action has a second background: critics claim that the Norwegian NIPT policy has led to a “divide” among women: “The introduction of NIPT has created a divide in which younger women, who have the lowest risk but the most pregnancies, have to pay an unnecessarily high price for the same testing that older women receive free of charge at their local hospital” (Glad 2022: n.p.). Glad describes this arrangement as “illogical” and advocates the provision of access to this form of care in *state-run* hospitals also for pregnant women under 35 years of age against a “fixed charge” (ibid.). Especially in rural regions, private NIPT centres are either not available at all or very hard to reach (ibid.), meaning that young women there have no equal access to health services” (ibid.): social justice thus does not exist.

### Regulating and monitoring new technologies: risks, ethical conflicts, and social disparities

The example of Norway highlights a principal dilemma of the way in which NIPT is used: a new technology with an essential focus on such a sensitive issue as nascent life and linked with far-reaching, conflict-ridden consequential decisions creates a complexity that is virtually impossible to grasp in its entirety. Decisions about frameworks, access rights, social equity, financial support, and quality control are interwoven at many different levels into a complex fabric that makes it difficult to predict potential impacts.

For would-be parents, these decisions involve new dimensions of responsibility that make them “gatekeepers” over lives to come. At the level of individual decisions, they contribute to an overall effect that has the potential to create a “sorting society” - even when the individual decision is not intended as such and is a reflection of an acutely distressful personal situation. Individual decisions are always, however, embedded in a social and political context and can thus be portrayed as “autonomous” only when this complex texture is blanked out; in this context, autonomy is understood as “relational autonomy” a concept that Seavilleklein (2019: 72) transferred to choices and decisions in the context of prenatal screening. In a relational concept of autonomy “persons are viewed as relational beings embedded within and shaped by a web of interconnected relationship” (ibid.). This web is made up in part of contextual factors such as political and economic aspects as well as governmental guidelines as exemplified in the case of Norway. It may also be perceived as general external pressure or influence from health professionals: a small percentage of women interviewed in the Netherlands about their experience with NIPT as primary screening for aneuploidy indicated that it was not their decision whether or not to have the test performed. “7% felt they were expected to choose NIPT and 6% reported that the midwife influenced their decision” (Kristalijn et al. 2022: 9).

New dimensions of responsibility are emerging at many levels, including for actors on the political stage and in professional societies: deciding on rules that are consistent with normative orientations and general legal conditions and that anticipate potential side-effects and risks has proven to be a difficult matter. In addition, the example of Norway illustrates how incentives from the market economy conflict with the requirements and framework conditions of state institutions and result in paradoxical consequences. From this viewpoint, risks, ethical conflicts, and social disparities appear almost inevitable. There are no control mechanisms available yet that avert harm from the users of NIPT technology and their families. This is explicitly evident from the current case regarding the “Safety Communication” of the Food and Drug Administration in den USA.

### Causa USA – the FDA warning in 2022

For years, everything seemed to be going well: the first NIPTs from the “land of opportunity” conquered the global market. The US-American global players possessed the patent rights and took care to secure their “intellectual property” in court (Baldus 2016). Further developments of the tests from the first products, which were directed at the most common trisomies, in the direction of analytical methods capable of analysing the entire genome expanded the range of potential applications. These now made it possible to test for even very rare trisomies and microdeletions. NIPTs were also increasingly used by women with low-risk pregnancies; in these areas, however, the validity of NIPTs is far less accurate, as discussed above.

As a consequence, women received test results that were subsequently found to be false; a problem that was already familiar in professional circles, but one that received broad public attention only after an investigative article was published in the New York Times (Kliff and Bhatia 2022) in January 2022. This research article alarmed the FDA into taking action (FDA 2022, Wise 2022).

Officially, there are no provisions for the regulation of the licensing and control of NIPTs by the FDA. This is due to the fact that NIPTs are classified as medicinal products from the category of so-called “Laboratory Developed Tests” (LDTs); as such, they are not subject to FDA control regulations. An interesting feature here, however, is that as early as 2012 the FDA considered stepping in to take regulatory action. At the time, the agency had a particular eye on the aggressive marketing strategies of the suppliers of the tests and was also concerned about the lack of any external validation of the NIPTs: “notably because of its aggressive marketing, including direct-to-consumer advertising, by companies and lack of ʻcomprehensive validationʼ” (Allyse et al. 2012: 3124, quoted after Ravitsky et al. 2021: 325). However, “this regulation has not materialized” up to the present day (ibid.). Although voices were raised that emphasized just how “critical” (ibid.) the regulation of NIPTs by the FDA is in consideration of “the sensitivity of the decisions that are made in the prenatal context” (King 2012, quoted after Ravitsky et al. 2021: 325), no further action was taken in the matter. A specific “threshold of accuracy” (ibid.) that was proposed as early as 2012 has so far been neither defined nor demanded. The tests have gained acceptance without any restrictions on their use and have since been expanded to cover the entire genome and also to detect very rare chromosomal disorders (rare autosomal trisomies, RATs) and microdeletions associated with low PPVs.

Ten years later, the FDA presumably regrets having issued mere declarations of intent at the time. In April 2022, following a spate of reported cases of false-positive test results some of which had fateful consequences, the agency saw itself forced to issue a warning. Designated as an “FDA Safety Communication” (FDA 2022), this warning expressly spotlights the risk of false-positive results yielded by genetic non-invasive prenatal screenings (NIPSs) and the inadequate validity of these tests, especially in the diagnostic search for RATs and microdeletions. In the same breath, the FDA takes care to emphasize its non-involvement: “the accuracy and performance of NIPS tests have not been evaluated by the FDA” (ibid.). In the same correspondence, however, the agency states its intention to work together with the US Congress to define the necessary legislation: “to establish a modern regulatory framework for all tests, including LDTs” (FDA 2022).

### Class-action lawsuits as a consequence

The publication of the investigative article in the New York Times not only sent shockwaves through the FDA, but also alerted users of the tests (Han 2022) and shareholders of the manufacturers (Barrons 2022). In first class-action lawsuits, the plaintiffs refer to the defects in the tests reported in the investigative article and the trauma they suffered as a result of using the tests, e.g. after receiving a false-positive result: “Due to the ʻHigh Riskʼ finding Plaintiff suffered emotional distress, stress, and anxiety throughout her pregnancy. Plaintiff ultimately gave birth to a healthy baby girl who did not suffer from any chromosomal abnormalities”. One of the aims of the lawsuit is to issue an injunction against the suppliers of the test manufacturers preventing them from marketing their test products under the claim that they are “reliable, accurate and offering peace of mind for patients regarding the viability of their pregnancies” (Han 2022).

Falsely positive findings also have an impact on women who opt for their pregnancy to be terminated following an anomalous test result, without waiting to undergo an invasive procedure to confirm the NIPT finding. They trusted the promises made by the manufacturers that their products are safe (Kliff and Bhatia 2022). In several documented cases, subsequent examinations showed that the finding was false, and that the unborn child would have been healthy. Even though these cases are only anecdotal evidence, they should still be cause for concern. The emotional distress and burdens placed on parents to be are high and should be prevented.

## Discursion: a gain of control or toxic knowledge?

By analogy to the claim made by Bschir (2018) that the “position of science is the result of a long control process”, the impact of prenatal testing on the courses of pregnancies can be interpreted as the result of control precepts and control experiments. Pregnant women are offered apparent control, which they accept and internalize. This is closely related to the particular phase of life and the fragile transition points of expectant parents, who have to make decisions within a limited period of time that decisively shape their future lives. Women during this period are a highly sensitive target group and are highly prepared to take responsibility and care for the unborn life. Narratives and appeals launched by the test industry suggestively permeate into this sense of responsibility and care, promising knowledge, security, and relief (Baldus 2018). That these narratives have now become the grounds for a claim filed by a deceived user possibly marks a turning point in the marketing and use of the NIPTs.

Knowledge has a very high priority in the modern-day information society; knowledge putatively makes autonomous subjects capable of making their own decisions in the first place, and not merely to resign themselves to a preordained biography as a matter of destiny. In the context of prenatal tests and diagnostic procedures, at first glance it appears a desirable and reasonable wish to gather as much knowledge as possible regarding the health status of the unborn child, so as to be able to take control over biographical twists of fate if necessary. Since the genetic anomalies that are sought out by the tests are not curable, the tests themselves have no therapeutic benefit for the future child. On the other hand, in rare cases they may be beneficial to the pregnant woman herself: if further investigation following a discordant NIPT result confirms a suspicion of maternal malignancy and the woman is treated at an early stage, it can be life-saving for her. This presupposes that any such incidental NIPT findings are routinely disclosed and lead to further management and treatment. However, this is not the case in all countries: “Thus, in some places in the world NIPT results suggestive of cancer are disclosed and influence post-test management, and in others they are not reported” (Benn et al. 2019: 341). Other benefits could involve the unborn child itself. New insights from prenatal genomics raise hopes that the early detection of monosomies such as Turner syndrome could lead to antenatal treatment of affected fetuses (Bianchi 2019). However, previous experience in European countries shows that the prenatal diagnosis of Turner syndrome is followed by termination of pregnancy in more than 50% of cases (EUROCAT). For other conditions such as Down syndrome, the rates are significantly higher (ibid.). What’s more, only a minute fraction of 3% of disabilities are due to genetic causes; most disabilities emerge in the course of a person’s lifetime as a result of disease (REHADAT 2021). The effort invested in prenatal identification of these 3% of disabilities can thus be questioned.

The number of unclear or inconsistent test findings increases in proportion to the scope of the spectrum of potential anomalies investigated by NIPTs or so-called microarray analyses. This places parents-to-be, but also consultants and physicians, before an ethical dilemma of far-reaching dimensions: how should they react to ambiguous information, what decision should they take on the further course of the pregnancy? In analogy to the mythological topos of “Pandora’s box”, Hashiloni-Dolev et al. (2019) speak of “Pandora´s pregnancy”: once the lid of the box is opened, previously unknown problems, concerns, and burdens emerge. Before using and implementing new technologies with a potential impact on nascent life, the important aspect is thus to find a balance between the “knowledge” that can be gained on the one hand and the options for action, the standards, and the values of a society on the other. The more “knowledge” that is gained that is inconclusive and opens up few or no curative therapeutic options at all, the less beneficial it is.

Bernhardt et al. (2013: 142) thus applied the term “toxic knowledge” to knowledge that is “uninterpretable or uncertain” (ibid.): knowledge that offers no clear prognoses and does not clearly state whether the detected genetic anomaly will at all become clinically manifest during the lifetime of the child-to-be. “When they were given such results, many women considered this information to be knowledge they wish they did not have” (ibid.). In retrospect, these women regretted the information they had received. Not only did they lose their joy of being pregnant, they also suffered fears, worries, and uncertainties well beyond the birth of their children (ibid.: 142, 143), even when the child was born without any defects. “Watchful waiting” became the norm during ongoing pregnancy, birth, and infancy” (ibid., 143). Knowledge can also unfold unpredictable effects (Gammon et al. 2018: 3): once it is out in the open, there is no putting it back in the box.

### Outlook

Examinations and tests made at the transition points along the path to parenthood should meet up to the highest quality and safety demands. It is astonishing that tests that are carried out in such a sensitive phase of a woman’s life as early pregnancy were able to make it to the market as laboratory developed tests without being subjected to control and evaluation by state agencies.

Thus, pregnant women serve as an “experimental collective” – with hitherto unknown consequences and an unclear risk-to-benefit ratio. While the tests were the subject of ethical debates and social controversies, these did nothing to prevent their implementation. The technology is progressing inexorably; the consequences, risks, and fears are borne first and foremost by the women and their partners: “NIPT is under debate because the benefits of detecting other fetal chromosomal aberrations must be balanced against the risks of discordant positives, parental anxiety, and a potential increase in (invasive) diagnostic procedures” (van der Meij et al. 2019: 1091).

Tests that can be simply and swiftly used to search for characteristics that contribute to the “othering” of human beings cannot be regarded isolated from their associative and ethical context. Describing them as harmless and risk-free negates this context. In terms of their potential impact on society, they are by no means harmless. The entire community can be understood as being affected – in ethical, economical, and health-related aspects. It is necessary to protect potential users against biased and smoothly worded marketing strategies and instead provide them with neutral, balanced, and officially monitored information (Nuffield Council on Bioethics 2017, Holloway et al. 2022). As the example of the FDA shows, this happens far too rarely. As long as the responsibility for ensuring that NIPT products are represented accurately and ethically rests with the manufacturers (Holloway et al. 2022) it will remain difficult to implement and monitor quality criteria. For example, the Advertising Standards Authority’s requirements in the United Kingdom that NIPT advertisements and brochures be presented in a manner that meets acceptable standards are not binding (ibid.: 50). This makes it even more important to install “market-access regulations of emerging molecular tests like NIPT and the claims made about them” (ibid.: 56).

In Europe, the so-called In Vitro Diagnostic Medical Devices Regulation (IVD Regulation) was enacted for the first time in 2017. It was then amended due to the COVID-19 pandemic by Regulation (EU) 2022/112, which extends the transition periods for products already on the market until 2025 or 2026. The aim of the IVD Regulation is to improve patient safety by introducing stricter procedures to prevent unsafe or non-compliant devices from ending up on the market (EUR-lex). Manufacturers are more clearly and more rigorously required to monitor the quality, performance, and safety of their products (ibid.). Nevertheless, it is still up to them to provide clinical evidence and reliability. Part of the IVD Regulation is that incidents must be reported to the relevant authority in the Member States. The term “incident” in this context is defined as “a malfunction, failure, or alteration of the characteristics or performance or incorrect labelling or instructions for use of a medical device that has, or may have, directly or indirectly resulted in the death or serious deterioration of the health of a patient, user, or other person” (Paul Ehrlich Institute). Thus, the critical factor monitored here is the safety of the health of the patient using the product, not the safety or accuracy of the results calculated by the product. The question arises whether the IVD Regulation will bring about a significant change for the monitoring of the quality of NIPTs.

When a nation has committed itself to upholding the reproductive rights of women while at the same time promising to protect the interests, rights, and dignity of people with disabilities, it has no choice but to take a clear stand at the intersection of conflicting arguments and disclose what interests are behind certain policies.

In China, the introduction of NIPTs did not involve “disability rights concerns” (Ravitsky et al. 2021: 329), nor does the use of the technology in that country stand in disagreement with the social code. Instead, in China the NIPT technology fits in perfectly with the ideological system and the plan for the prevention of disabilities: “In China, NIPT coincides with a national plan for disability prevention” (ibid.).

While models for the implementation of NIPT in the United States and Europe do not officially fall into line with plans for the prevention of disabilities, they do contribute towards making the early-stage genetic search for anomalies in the unborn child a normal practice, as a standard and routine method in which participation and compliance are desired.

Certain critical questions remain unresolved in these countries too: how earnestly does a society actually tackle the issue of the inclusion of people who deviate from the norm and have a special need for care? What measures do professional societies, associations, and regulatory authorities take to ensure that pregnant women are protected from harm, that there is an adequate provision of qualified professionals who give unbiased and comprehensive advice and information during pregnancy, at the same time addressing ethical issues and social implications? And how can people’s decision-making autonomy and their “right not to know” be practically implemented, at the same time counteracting the indirect discrimination of people with disabilities (Perrot and Horn 2022)?

Bio-power, as Schidel (2020) analyses in analogy to Foucault, in western countries of the 21st century no longer establishes itself “via institutional constraints” (Schidel 2020: 255); it nevertheless exists and unfolds its effect at an individual level: “It takes hold in the individual person by forcing the person, apparently of his own free will, to adjust to the norms, which are predefined as normality” (ibid.).

An interesting aspect in this context are reflections on how responsibility is identified and delegated within the “apparatus of choice” (Mills 2017, quoted after Stephenson et al. 2017). “Drawing on Foucault’s conception of an apparatus, Mills proposes that the ‘‘apparatus of choice’’ describes a relatively cohesive and coherent amalgamation of material and discursive elements that shape but do not determine practice in a given context, the central and organising characteristic of which is the notion of individual choice” (ibid.: 78). In their empirical study combining field observations in ultrasound settings with interviews, Stephenson et al. (2017) show how the emphasis on individual choice by patients framed as autonomous subjects can detract from conflicting roles and positions. This is, for example, the “dual positioning of pregnant women as both autonomous patients making decisions about their antenatal care and as members of a population whose participation has a collective purpose and benefit” (ibid.). From the scientific perspective, the question can be asked as to how the “institutional implementation of a certain type of responsibility” (Bschir 2018) is accomplished. Transposed to the area of prenatal diagnostics, the following analysis can be drawn: there is a clear division of the roles between the individual actors involved. While the manufacturers of the tests and the physicians view themselves as producers of knowledge and providers of information, acting neutrally, objectively, and non-judgmentally, the women are assigned the role of the self-responsible actors on the basis of the principles of self-determination, reproductive freedom, and informed consent. This model of the assignment of responsibilities fully disregards the complex matrix of social interrelationships, interdependencies, and commercial interests. In this way, selective decisions are decontextualized and individualized. The multiwoven texture of medical feasibilities, social expectations, social norms, and individual problems is reduced to a private momentum.

In addition to the ‘apparatus of choice’ it is enlightening to consider the ‘apparatus of normalization’ that “‚merely‘ call upon us to orient our behavior to that which the majority demands of each of us“ (Waldschmidt 2015, 194 f. quoted after Schidel 2020: 255). Ultimately, many individually justified decisions taken against leading a life with a disabled child result in a normalization of a structure of values that “at the societal macrolevel constitute a form of structural discrimination” (Schidel 2020: 253) and thus further cement the exclusion of people with disabilities (ibid.). According to Schidel, norming and normalization processes in the field of biopolitics can form a multitude of structural discriminations. She therefore argues that socially powerful conceptions of norms must be critically questioned again and again and deconstructed for their exclusionary effect (ibid.: 260).

In this respect, prenatal selection can be seen as a modernized form of exclusion, brought forward into the prenatal sphere. The “modernization” is based on three central developments:

1. the abstinence of coercion;
2. the delegation of responsibility by the paradigm of freedom of choice; and
3. the economization and individualization of the range of health care by ‘optional services’ (cf. Baldus 2006: 55).

The task of resisting predefinitions of normality and an insidious routinization of prenatal selection and of developing alternative concepts cannot be delegated. It is a task for society as a whole, one that cannot be left to ethics committees, regulatory authorities, or medical societies alone. This notwithstanding, these instances bear the responsibility for the creation of a framework that gives the actors involved a sense of security and that supports parents-to-be in their decisions. It is precisely because non-invasive prenatal tests are depicted as being so simple, safe, and harmless that a clearly defined awareness regarding their use must be created. This clarity emerges with the question as to how we want to shape society and what space people with disabilities have in this society.
